# The Treatment of Sarcoma

**Published:** 1912-05-04

**Authors:** 


### THE TREATMENT OF SARCOMA.

### The Use of Coley's Fluid in Inoperable Cases.

The fact that certain patients suffering from sarcoma had undergone spontaneous cure after an attack of erysipelas led Dr. Coley, of New York, to endeavour to give erysipelas to patients suffering from inoperable sarcoma in the hope that this natural mode of cure might be imitated. This was in 1891, and the early experiments were disappointing. It was found difficult to give people erysipelas, and it was also found that the injection of living cultures of the streptococcus of erysipelas was sometimes followed by a fatal result. In spite of all this, however, the important fact was established that the injections did, to a certain extent, inhibit the growth of sarcomatous tumours. Killed cultures were then substituted for the living organisms and were found to be equally effective.

In the following year (1892) Rodger, of Paris, discovered how greatly the virulence of the streptococcus of erysipelas could be enhanced by growing with it the *Bacillus prodigiosus*. Coley seized upon this fact and injected his patients with a mixture of the killed cultures of the two organisms. He then found that he had produced a powerful therapeutic agent, and in his hands many cases of sarcoma have been treated with complete success. *B. prodigiosus* is grown on agar for ten days, the thick red growth is then scraped off and rubbed up in a mortar with physiological saline solution. It is then added to a three weeks' cultivation of streptococcus in broth, both cultures having been previously sterilised by heat. After mixing and bottling the toxins, they are again subjected to a temperature of 75°.6 for two hours as a further precaution. The fluid so prepared is highly toxic, and it is necessary to proceed with extreme caution. No more than ¼ minim should be used as an initial dose, and this may be injected either into the tumour—the preferable proceeding—or into the muscles of the buttock or elsewhere. Even with so small a dose a reaction frequently follows; but if not the dose is increased daily by ¼ minim until a well-marked reaction is obtained. This is characterised by a high temperature (102° to 105° F.)—possibly accompanied by a rigor—nausea or even vomiting, frontal headache and pains in the limbs. The temperature reaches its maximum about four hours after the injection and as quickly falls, so that by the following day the patient is again ready for another dose. The writer has found that by giving the injections about 4 to 5 p.m., the patient gets his bad time over in the evening and has a comparatively undisturbed night's rest, and is able to get up for a little while the following morning. It is important that the patient should remain in bed during the twelve hours or so following the injection. In a few cases the discomfort and exhaustion resulting from the treatment is so considerable that it has to be temporarily suspended.

If the usual precautions as to sterilising the hypodermic needle and the skin of the patient be attended to, no local disturbance at the site of the injection is to be apprehended. However, when the growth is very vascular and on the point of breaking down, the local action of the mixed toxins appears to hasten the necrotic process. In such cases it is, therefore, wise to make the injections in parts remote from the tumour.

In favourable cases, *i.e.* in those cases where any curative action is apparent, a change in the condition of the tumour usually becomes manifest during the second week of treatment: the growth becomes smaller, less vascular and more movable, and may ultimately completely disappear. The number of cases in which this has occurred is, of course, not large; Dr. Coley has himself treated some four hundred cases of sarcoma, and states that in 10 per cent. he has obtained complete disappearance of the tumour; and this, be it remembered, in cases which in the majority of instances were really beyond the scope of surgery. How or why Coley's fluid exerts this influence it is only possible to conjecture. All that is definitely known is that injection of the mixed toxins brings about a very considerable leucocytosis, and at the same time a certain amount of coagulative necrosis of the tumour. It is now widely believed that, at any rate some kinds of sarcomata are of parasitic origin: the very fact that certain of them do undoubtedly disappear after the injection of Coley's fluid favours this view of their nature. It is conceivable that the use of the toxins induces the formation of anti-bodies, which are "anti," not only to the streptococcus and *B. prodigiosus*, but also a variable extent to the parasite causing sarcoma.

To turn to a consideration of the kinds of case most amenable to the toxin treatment. In the first place, none but cases of sarcoma should be treated, in carcinoma the toxins are quite valueless. Again, it is always important to exhaust every method of clinical diagnosis, including, whenever possible, the removal of a piece of tissue for histological examination, before the trial of the treatment is commenced. With this proviso any case of sarcoma may be undertaken, for Dr. Coley has had success with such varied conditions as round and spindle cell sarcoma of bone, sarcoma of the abdominal wall and of the tonsil. During last year in this country two completely successful cases were published, one by Spencer—a sarcoma of the abdominal wall, and the other, a growth of the humerus, by Ashdown.

There are two other aspects of Coley's treatment that still require consideration. It sometimes appears that a sarcomatous mass after improving up to a certain point remains stationary. But even modified success of this kind may be of the utmost value, for a tumour originally fixed and altogether inoperable, may have become so altered as to bring it within the range of justifiable surgery and so permit of its successful removal. Secondly, Coley's fluid should undoubtedly find an important place as a prophylactic measure after operations for sarcoma, with a view to obviating future recurrence. When used for this purpose the dose need not be pushed further than is necessary to obtain a rise of temperature to 99° or 100°, accompanied by slight muscular pains. The injection should be repeated two or three times a week and continued for two or three months. In conclusion, it is necessary to emphasise the fact that the toxins are not suggested as an alternative to surgical treatment in suitable cases. Coley himself only advises their use for inoperable sarcoma, or in such cases as periosteal sarcoma of the femur, in which recurrence after operation we now know to be the invariable rule.

